# Gut virome profiling identifies an association between temperate phages and colorectal cancer promoted by *Helicobacter pylori* infection

**DOI:** 10.1080/19490976.2023.2257291

**Published:** 2023-09-25

**Authors:** Shiqi Luo, Jinlong Ru, Mohammadali Khan Mirzaei, Jinling Xue, Xue Peng, Anna Ralser, Raquel Mejías-Luque, Markus Gerhard, Li Deng

**Affiliations:** a Institute of Virology, Helmholtz Centre Munich — German Research Centre for Environmental Health, Neuherberg, Germany; b Chair for Preventions of Microbial Diseases, School of Life Sciences, Technical University of Munich, Freising, Germany; c Faculty of Biology, Biocenter, Ludwig Maximilian University of Munich, Munich, Germany; d Institute for Medical Microbiology, Immunology and Hygiene, Technical University of Munich, Munich, Germany; e German Center for Infection Research (DZIF), Munich Partner Site, Munich, Germany

**Keywords:** Colorectal cancer, helicobacter pylori, temperate bacteriophage, auxiliary metabolic genes, bacteria-phage interaction

## Abstract

Colorectal cancer (CRC) is one of the most commonly diagnosed cancers worldwide. While a close correlation between chronic *Helicobacter pylori* infection and CRC has been reported, the role of the virome has been overlooked. Here, we infected *Apc*-mutant mouse models and C57BL/6 mice with *H. pylori* and conducted a comprehensive metagenomics analysis of *H. pylori*-induced changes in lower gastrointestinal tract bacterial and viral communities. We observed an expansion of temperate phages in *H. pylori* infected *Apc*^*+/1638N*^ mice at the early stage of carcinogenesis. Some of the temperate phages were predicted to infect bacteria associated with CRC, including *Enterococcus faecalis*. We also observed a high prevalence of virulent genes, such as *flgJ*, *cwlJ*, and *sleB*, encoded by temperate phages. In addition, we identified phages associated with pre-onset and onset of *H. pylori*-promoted carcinogenesis. Through co-occurrence network analysis, we found strong associations between the viral and bacterial communities in infected mice before the onset of carcinogenesis. These findings suggest that the expansion of temperate phages, possibly caused by prophage induction triggered by *H. pylori* infection, may have contributed to the development of CRC in mice by interacting with the bacterial community.

## Introduction

Colorectal cancer (CRC) is one of the most prevalent malignancies in the world, with low survival rates.^1^ Multiple factors including genetics, lifestyle, and an altered microbiome are known to play a role in CRC, and recent studies have suggested a potential link between *Helicobacter pylori* infection and the development of the disease.^2^ More than half of the world’s population carries *H. pylori* in the upper gastrointestinal tract, and colonization is often asymptomatic.^3^ Chronic infection with this bacterium leads to gastric inflammation and may even induce gastric cancer.^4^ In addition, increasing epidemiological studies have shown a close correlation between *H. pylori* infection and CRC.^2,5^

It has been shown that *H. pylori* infection contributes to tumorigenesis by deregulating gastrointestinal immunity and activating oncogenic signaling pathways in the gastrointestinal tract.^6–8^ In addition, the infection significantly alters not only the gastric microbiota^2,9,10^ but also distant, intestinal microbial communities.^11^ We recently reported a unique *H. pylori*-driven immune alteration signature characterized by a reduction in regulatory T-cells and proinflammatory T-cells, as well as *H. pylori* induced pro-carcinogenic STAT3 signaling, loss of goblet cells and increase of mucus Altogether, it was shown that *H. pylori* infection contributes to the development of CRC by disrupting gut homeostasis.^12^

While changes in the gut bacterial community are associated with CRC, the role of gut viruses remains largely unexplored. Bacteriophages, or phages, represent the vast majority (97.7%) of the gut viruses in healthy western adults,^13^ and their constant interactions with their bacterial hosts shape gut microbial communities and maintain gut homeostasis.^14^ In addition to regulating the bacterial communities by specifically eliminating their bacterial hosts, phages influence the gut ecosystem by directly interacting with the immune cells and modulating the host immune response.^15^ Recent studies have observed significant changes in the fecal virome, especially the phage population, in CRC patients.^16–19^ In addition, the expansion of phages in a mouse model led to intestinal inflammation and colitis by activating IFN-*γ* through a TLR9-dependent pathway, which exacerbated colitis in the model.^20^ Further, phages transfer the auxiliary metabolic genes (AMGs), which are prevalent in gut phage genomes,^21–23^ to and between bacterial genomes through horizontal gene transfer (HGT).^24^ These genes are largely involved in bacterial cellular processes and extracellular virulence,^25,26^ thus play a significant role in human health and diseases by altering bacterial metabolism.^15^

Here we aim to study the role of gut viruses using a mouse model of *H. pylori*-promoted CRC.^12^ We used shotgun viral metagenomic sequencing to characterize the viral community composition of the cecum, stool and intestinal samples from i) *Apc*^*+/1638N*^ and ii) C57BL/6 mice, with and without *H. pylori* infection, as well as at different time points (before the onset of colonic tumors and afterward). We identified phage-encoded AMGs related to carcinogenesis. Finally, we studied the correlations between the changes in the viral communities and the bacterial communities with CRC development.

## Results

### *H.* *pylori* infection alters the gut viral community and expands temperate phages in *Apc^+/1638N^* mice

**Figure 1.**
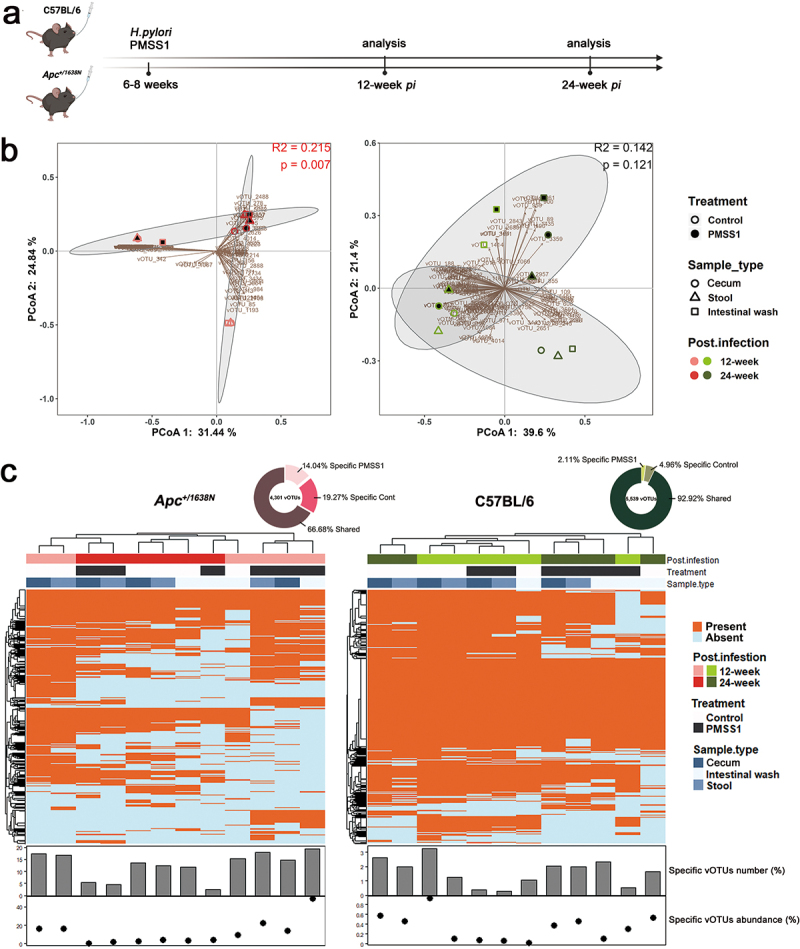
Altered gut virome in *Apc*^*+/1638N*^ mice with *H. pylori* PMSS1 infection. (a) Animal setting in the study. (b) Principal coordinate analysis (PCoA) of Bray-Curtis distance of virome. Each dot represents pooled samples from three to five mice. Treatment groups are indicated respectively. The 95% confidence intervals of the control and *H. pylori* PMSS1 groups are shaded accordingly. (c) Distribution of shared and specific vOTUs: 4,301, 5,539 detected vOTUs in *Apc*^*+/1638N*^ and C57BL/6 mice were assigned into two categories as shared vOTU, specific vOTU (to PMSS1 infection and to control). The proportion of numbers and the relative abundance of specific vOTUs in each sample. The scales on the y-axis are optimized to present the data. One dot/bar represents samples from three to five mice.

To investigate the impact of the virome and bacteriome on the development of extra gastric cancer promoted by *H. pylori* infection, tumor-prone *Apc*^*+/1638N*^ and wild-type C57BL/6 mice were infected with *H. pylori* for 24 weeks (Figure 1a). We observed a significant increase in tumor number in the small intestine of *H. pylori*-infected *Apc*^*+/1638N*^ mice at 12 weeks (Supplementary Figure S1a-c),^12^ which further increased at 24 weeks post-infection (*pi)* as shown in previous study.^12^ Colonic tumors were only observed 24 weeks *pi* in *Apc*^*+/1638N*^ mice.^12^ These data indicated that the carcinogenesis process was initiated earlier in *H. pylori*-infected mice (at 12 weeks *pi*), and thus defined this period as the pre-onset stage of CRC.

We characterized the bacterial and viral communities of the cecum, stool and intestine from both control and *H. pylori*-infected mice sacrificed 12- and 24 weeks *pi*. We identified a total of 3,649 unique amplicon sequence variants (ASVs) for the bacterial community. However, we did not observe a significant alteration in the bacterial composition, diversity and richness caused by *H. pylori* infection (Supplementary Figure S2).

In addition, we identified 5,687 unique viral operational taxonomic units (vOTUs) from these samples. We observed lower viral diversity and richness in *Apc*^*+/1638N*^ compared to C57BL/6 mice regardless of *H. pylori* infection (Supplementary Figure S3a), emphasizing the importance of host genetics in shaping the viral communities. *H. pylori* infection reduced the virome diversity and richness in samples from C57BL/6 mice throughout the study, while it reduced the virome richness in *Apc*^*+/1638N*^ mice only at 12 weeks *pi* (Supplementary Figure S3a). Yet, the changes in the virome composition were more pronounced in *Apc*^*+/1638N*^ compared to C57BL/6 mice (Figure 1b).

We further characterized changes in the virome associated with *H. pylori*-promoted intestinal cancer. More specifically, we analyzed the presence or absence of different vOTUs in samples from *Apc*^*+/1638N*^ and C57BL/6 mice with and without *H. pylori* infection. We identified many vOTUs which were only present in infected mice: 14.04% of 4,301 in infected *Apc*^*+/1638N*^ mice and 2.11% of 5,539 in infected C57BL/6 mice (Figure 1c). Therefore, *H. pylori* infection altered the virome more strongly in *Apc*^*+/1638N*^ mice than in C57BL/6 mice (Figure 1b). In addition, vOTUs specific to *H. pylori* infection accounted for a larger proportion of viral communities in infected *Apc*^*+/1638N*^ mice at 12-week *pi* (14.20% in cecum, 22.31% in stool, 49.16% in intestinal wash) than that at 24-week *pi* (0.37% in cecum, 1.48% in stool, 4.06% in intestinal wash) (Figure 1c). This suggests more significant changes in viral communities before the onset (12-week *pi*) than after the onset of *H. pylori*-promoted CRC (24-week *pi*).

**Figure 2.**
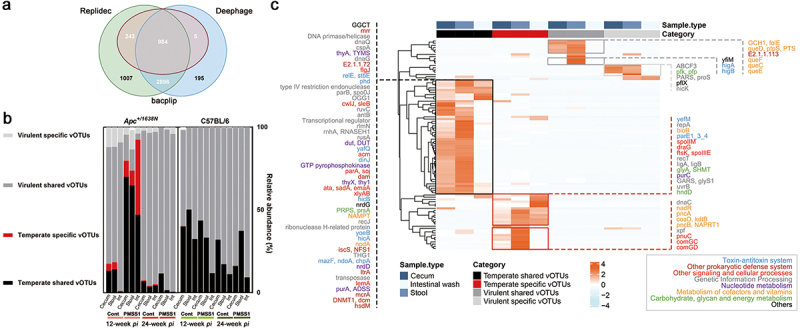
Expanded temperate vOTUs, and specific vOTUs in infected *Apc*^*+/1638N*^ mice carrying crucial AMGs in the pre-onset of *H. pylori*-promoted CRC. (a) Temperate vOTUs determination by three tools: BACPHLIP,^27^ Deephage^28^ and Replidec.^29^ a vOTU is assigned to be temperate when agreed by at least two tools. (b) The ratio of temperate specific (red, the temperate vOTUs presented in either control or infected mice), temperate shared (black, the temperate vOTUs presented in both control and infected mice), virulent specific (light gray, the virulent vOTUs presented in either control or infected mice) and virulent shared vOTUs (dark gray, the virulent vOTUs presented in both control and infected mice) in gut virome of *Apc*^*+/1638N*^ and C57BL/6 mice. (c) Heatmap shows the AMGs, overrepresented among significantly abundant temperate shared, temperate specific, virulent shared and virulent specific vOTUs in infected mice at 12-week *pi*. Functions of these AMGs were annotated using DRAM-v and UniProtKB.

The majority of viruses found in the samples were phages that belonged to three classes: *Caudoviricetes*, *Malgrandaviricetes*, and *Faserviricetes*, which made up approximately 97.88% ± 3.60% on average (Supplementary Figure S4b). This aligns with previous research indicating that phages are the most prevalent viruses in the human gut.^13^ We also predicted the replication cycle of these viruses or vOTUs using BACPHLIP, Deephage and Replidec and identified 4,128 vOTUs (72.59%) as temperate phages (Figure 2a). Interestingly, the relative abundance of temperate phages (79.78% in cecum, 73.79% in stool and 92.49% in intestinal wash) was high in *H. pylori*-infected *Apc*^*+/1638N*^ mice at 12-week *pi*, while virulent phages were dominant at 24-week *pi* (88.19% in cecum, 98.33% in stool and 99.36% in intestinal wash) (Figure 2b). This suggests a higher probability for phage-mediated HGT in *H. pylori*-infected *Apc*^*+/1638N*^ mice at 12-week *pi* relative to at 24-week *pi*.

## Prevalence of temperate phages and auxiliary metabolic genes precedes the onset of *H. pylori*-promoted CRC

We next predicted the host range of significantly abundant vOTUs (Sig.vOTUs: please see the material and method section for more details about viral groupings; Supplementary Figure S4 and S5, Supplementary Table S1 and S2) in *H. pylori*-infected *Apc*^*+/1638N*^ mice to investigate the potential role of temperate vOTUs in tumorigenesis. We found several vOTUs that belong to the *Caudoviricetes* class that infect bacteria known to either prevent or promote colorectal cancer (CRC). The formers include *Lactobacillus gallinarum*,^30^ *Lactobacillus kefiranofaciens*,^31^ *Lactobacillus acidophilus*,^32^ and *Alistipes shahii*.^33^ On the other hand, some vOTUs infect bacteria associated with promoting CRC, such as *Alistipes onderdonkii*,^34^ *Alistipes finegoldii*,^35^ *Flavonifractor plautii*,^36^ and *Enterococcus faecalis*, which has been linked to invasive phenotypes of colon cancer cells.^37^ Interestingly, the vOTUs infecting *E. faecalis* were highly abundant in samples from infected mice at 12-week *pi* (Supplementary Figure S4a-b, and S5) while vOTUs infecting *Alistipes spp*. increased in abundance after 24 weeks (Supplementary Figure S5a-c). These findings suggest that there is a complex phage-bacterial infection network associated with *H. pylori*-promoted CRC, where temperate phages play a key role in the progression of CRC (Supplementary Figure S4b and S5b).

To further understand the functional role of temperate vOTUs at 12-week *pi* and their contribution to CRC, we compared the abundance of AMGs in Sig. vOTUs of the samples from 12- (Figure 2c) and 24-week *pi* (Supplementary Figure S6). We observed a higher abundance of AMGs in temperate vOTUs isolated from infected *Apc*^*+/1638N*^ mice at 12-week *pi* (Figure 2b) compared to 24-week *pi*, suggesting a more significant contribution of temperate phages to early carcinogenesis. In addition, the abundant AMGs encoded by Sig. vOTUs from the infected *Apc*^*+/1638N*^ mice collected at 12-week *pi* were more diverse than those at 24-week *pi* (94 vs. 45 AMGs) (Figure 2c and Supplementary Figure S6). In addition, 65.96% of the 94 AMGs were found abundant in temperate vOTUs from the infected *Apc*^*+/1638N*^ mice at 12-week *pi* (Figure 2c). Then, we annotated these AMGs using DRAM-v^38^ and UniProtKB (Supplementary Table S3 and S4), they mainly encode genetic information processing, signaling and cellular processes, metabolism, etc. (Figure 2c). Several AMGs were only detected in temperate vOTUs (Supplementary Table S3). These include toxin-antitoxin systems, which are linked to bacterial cell growth and death.^39^ In addition, peptidoglycan hydrolase (*flgJ*) and N-acetylmuramoyl-L-alanine amidase (*cwlJ, sleB*) were also detected, which are associated with bacterial cell growth and mobility by affecting the survivability of their bacterial hosts.^40,41^ Furthermore, the presence of specific transporters, including trimeric autotransporter adhesin (*ata, sadA, emaA*), which plays a critical role in the virulence of bacteria,^42–44^ was found to be more prominent in temperate vOTUs than in virulent vOTUs. These genes can be potentially transferred through phage-mediated HGT to bacteria, expand their virulence, and contribute to CRC development or progression.

## Phage-bacteria co-occurrence network supports the strong phage-bacteria linkages prior to the onset of *H. pylori*-promoted CRC

**Figure 3.**
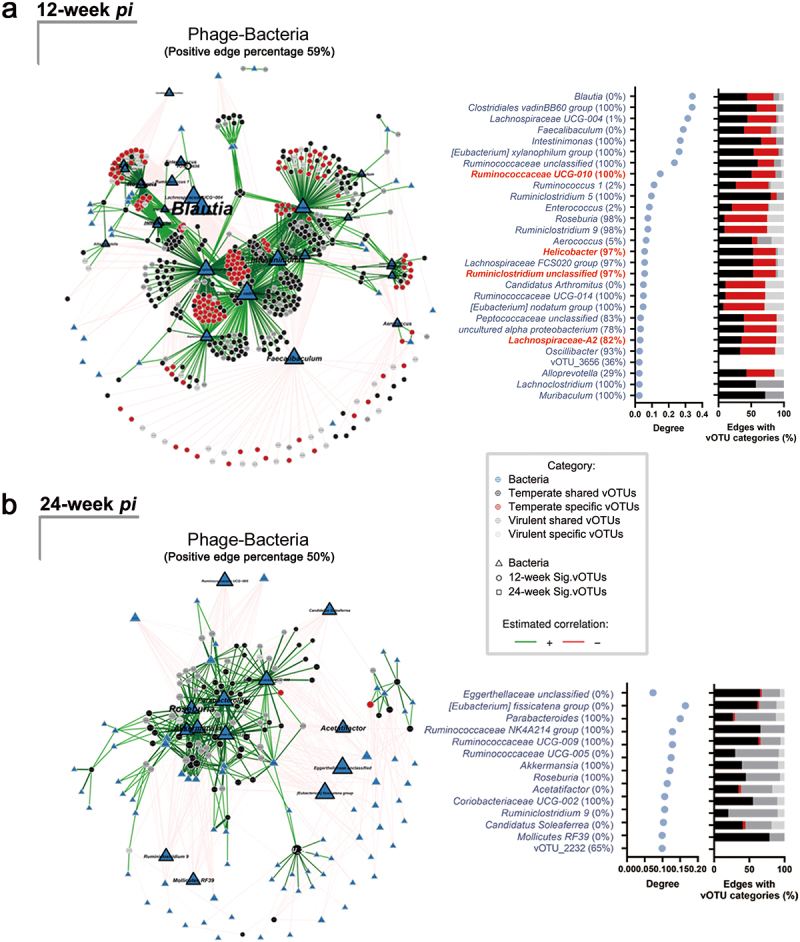
Network of significant abundant vOTUs and bacterial community is centered at *Ruminiclostridium, Ruminococcaceae* and *Helicobacter* during the pre-onset of *H. pylori-*promoted CRC. (a) Network of 494 curial vOTUs related to the pre-onset of *H. pylori*-promoted CRC and bacterial communities and the degree centrality measures in control and *H. pylori*-infected *Apc*^*+/1638N*^ mice at 12-week *pi*. (b) Network of 92 Sig. vOTUs related to the onset of *H. pylori*-promoted CRC and bacterial communities and the degree centrality measures in control and *H. pylori*-infected *Apc*^*+/1638N*^ mice at 24-week *pi*. The node sizes reflect their degree of connection, those with a degree value above the empirical 95% quantile and are identified as hub taxa in the network of phage and bacteria in mice at 12- and 24-week *pi* (in navy). Hub taxa in both networks of phage and bacteria, and bacterial taxonomy are highlighted in red. Percentage of positive edges of each node is shown in the name of the Y-axis. The percentage of edge linked with Sig. vOTUs from four vOTU categories for bacteria is illustrated.

To explore the effect of phage-bacteria and bacteria-bacteria interactions at different stages of CRC, we constructed multiple association networks for samples from *H. pylori*-infected *Apc*^*+/1638N*^ mice and controls at 12- and 24-week *pi* (Figure 3 and Supplementary Figure S7). We observed dissimilarities in the topologies of bacteria-bacteria and phage-bacteria networks between 12- and 24-week *pi*, suggesting different interaction patterns in pre-onset and onset of CRC.

Multiple interaction networks with potential roles in CRC development were observed. For example, in *H. pylori*-infected samples from 12-week *pi*, we found correlations between *Ruminiclostridium* unclassified, *Ruminococcaceae UCG-004*, *Ruminococcaceae UCG-010*, and *Helicobacter*, as the hub taxa in the community (Supplementary Figure S7b). This is especially interesting since *Ruminiclostridium* and *Ruminococcaceae* have been previously linked to the development of CRC.^45,46^

Moreover, we found a correlation between vOTUs and specific bacterial taxa in the network. The bacterial taxa that were hubs in both phage-bacteria and bacteria-bacteria networks (Figure 3a and Supplementary Figure S7b) surprisingly revealed the association between specific temperate vOTUs and hub taxa *Ruminiclostridium* unclassified (34.38%), *Ruminococcaceae UCG-010* (36.47%), *Helicobacter* (34.38%) (Figure 3a and Supplementary Table S5). In addition, we found that the specific temperate vOTUs were associated with a relatively higher number of hub taxa when considering their smaller proportion. These accounted for 28.54% of the significantly abundant vOTUs but contributed to 36.76% of the total phage-bacteria network (Supplementary Table S5).

However, we did not find any shared hub bacteria (Figure 3b and Supplementary Figure S7d) in the samples collected at 24-week *pi*. *Helicobacter* was not the hub taxa in the bacterial community in infected mice (Supplementary Figure S7d). Only four hub bacteria in the phage-bacteria network (*Ruminococcaceae UCG-004*, *Ruminococcaceae UCG-005, Ruminococcaceae UCG NK4A214* and *Acetatifactor*) showed a high eigenvector centrality within the bacterial community in infected mice (Supplementary Figure S7d). Furthermore, our data revealed that the phage community at 12-week *pi* exhibited strong associations with bacterial taxa that have previously been linked to CRC, such as a decrease in *Blautia*,^47^ and *Faecalibaculum*,^48^ as well as an increase in *Intestinimonas*^49^ (Figure 3a). However, we did not observe significant differences in the relative abundance of the phages infecting these taxa among different treatments (Supplementary Figure S8). Our results indicate that there are strong phage-bacteria interactions during the pre-onset of *H. pylori*-promoted CRC.

## Discussion

Colorectal cancer is the third most common cancer worldwide.^1^ Several risk factors, including *H. pylori* infection, are known to play a role in the development of the disease.^12,50^ Although most *H. pylori* infections stay asymptomatic, chronic infections lead to gastric inflammation and cancer.^4^ Phages play a significant role in maintaining gut homeostasis and are hypothesized to induce oncogenesis by altering bacterial community composition and physiology.^15,17,51^ In addition, alterations in the fecal virome have been linked to CRC,^16,19,51^ yet more mechanistic studies are needed to explore whether they play a *causative role*. Here, we studied gut viral community composition of *H. pylori*-promoted colorectal cancer in tumor-prone *Apc*^*+/1638N*^ compared with wild-type C57BL/6 mice, and found significant changes from *Apc*^*+/1638N*^ mice in 12 weeks *pi* which reflects the early stage of CRC.

Specifically, we observed differences in gut viral communities in samples from *H. pylori*-infected mice compared to non-infected controls. We found a decrease in diversity of the viral communities through the study period in C57BL/6 mice, and a reduction in richness of these communities after 12 weeks of *H. pylori* infection in *Apc*^*+/1638N*^ mice. In addition, the abundance of vOTUs specific to *H. pylori* was higher at 12 weeks compared to 24 weeks post-infection. The significance of these changes was greater at 12 weeks post-infection than at 24 weeks, which suggests a stronger link between the alteration of these communities and tumorigenesis at earlier stage of CRC development.

The majority of the identified viruses were phages, belonging to *Caudoviricetes*, *Malgrandaviricetes*, and *Faserviricetes* classes. Interestingly, most of the expanded phages were temperate phages that can replicate either lysogenically as prophages or lytically by producing progeny. This is also consistent with previous studies that have reported an increase in temperate phages in patients with inflammatory bowel disease (IBD).^52^ In addition, an association between temperate phages and CRC have been previously reported.^19^ Lysogens or bacteria with integrated prophages in their genomes have been observed in high abundance in the murine gut,^53^ and it has been suggested that inflammatory events may initiate prophage induction in these bacteria by activating oxidative stress and triggering an SOS response in bacteria.^54^

Previous studies have shown that *H. pylori* infection causes immune and epithelial alterations in the intestinal tract by reducing regulatory T-cells and proinflammatory T-cells, inducing pro-carcinogenic STAT3 signaling and a loss of goblet cells.^12,55^ While these changes promote colorectal carcinogenesis in mice, they can also cause prophage induction, which could explain the expansion of temperate phages upon *H. pylori* infection in our study. This is specifically important as prophage induction is suggested to exacerbate disease severity in dysbiosis-associated diseases such as IBD, through immune responses triggered by bacterial lysis and released phage progeny.^52,56^ Similarly, the induction of prophages triggered by *H. pylori* infection could have contributed to CRC development in mice. More specifically, cell lysis due to prophage induction could release pathogen-associated molecular patterns (PAMPs) and danger-associated molecular patterns (DAMPs), which could trigger the release of proinflammatory cytokines, aggravating intestinal inflammation and dysbiosis.^57^ In addition, cell death by prophage induction in commensal bacteria reduces their protective effects against pathobionts.^58^ The induced phages could also trigger immune responses, including the production of IL-12, IL-6, IL-10, and IFN-γ-induced protein 10,^59^ which have been associated with CRC.^60^ Our host range predictions show that the expanded phages can target a wide diversity of bacterial taxa with distinct functions in gut health, including species with an anti-tumor effect like lactic acid-producer *Lactobacillus* and those associated with tumorigenesis such as *E. faecalis*, suggesting a general induction pattern in gut bacteria by *H. pylori* infection regardless of their taxa. The induction of prophages can significantly alter bacterial community composition in the gut, resulting in dysbiosis and horizontal gene transfer among bacteria with an unpredicted impact on gut health. Temperate phages, in general, encoded more AMGs than virulent phages, which is consistent with their potential role as contributors to bacterial fitness in the gut.^61,62^ In addition, our data indicated a higher abundance and diversity of AMGs in mice 12 weeks after infection with *H. pylori*, thus at the early stage of CRC development. The high prevalence of virulent genes such as *flgJ* and *cwlJ, sleB* in these phages and the possibility of temperate phages infecting taxa such as *E. faecalis* which is associated with CRC development further strengthens the hypothesis that they are involved in colorectal carcinogenesis. However, the underlying mechanisms remain to be explored.

We also found a positive correlation between *H. pylori* infection and an increased linkages between phages and *Ruminiclostridium* and *Ruminococcaceae*, two cancer-related bacterial genera.^45,46^ Interestingly, these changes were observed only 12 weeks after *H. pylori* infection, suggesting that they are mainly involved in the early stage of tumorigenesis while following a “driver passenger model”- the driver (*H. pylori*) causes changes in the tumor microenvironment enabling the expansion of passengers (cancer-causing taxa) that promote disease development.^63^ These changes were only observed in tumor-prone *Apc*^*+/1638N*^ and not wild-type mice, suggesting the importance of genetics in host-microbe interactions and colorectal carcinogenesis.

Our study has shed light on the effect of *H*. *pylori*-specific alterations in gut homeostasis and its knock-on effect on the gut virome and provides evidence of the role of temperate phages in the early stage of CRC development. We believe that the interactions between phages associated with CRC, the gut bacterial community, and host immunity has potentially impacted the disease progression, both directly and indirectly. Specifically, these phages may have changed cancer cell behavior by altering the gut bacterial community, interacting with immune cells, or transcytosing into colonic epithelial cells.^64^ It is also possible that tumor cells have altered the composition of these viruses by secreting tumor-associated factors and interacting with the immune system and gut bacteria. However, to fully understand the role of phages in CRC and how they affect its development, it is essential to perform functional analyses of these viruses in the human gut.

Given the high prevalence of both lysogens and *H. pylori* in the gut, these findings could have potential implications for controlling *H. pylori-promoted* CRC. However, going forward, it will be necessary to further validate our results by assessing the effect of these phages on CRC development independent from *H. pylori* infection. It is also essential to investigate whether these findings are specific to *H. pylori* infection or if other gut pathogens can also stimulate the expansion of temperate phages, and how this would impact gut homeostasis.

## Materials and methods

### Study design and sample collection

*Apc*^*+/1638N*^ mice were provided by Prof. Klaus-Peter Janssen (Klinikum rechts der Isar, Munich) and bred under specific pathogen-free conditions in the animal facility at the Technical University of Munich. C57BL/6 mice were purchased from Envigo RMS GmbH and acclimatized to the animal facility for 1–2 weeks as controls for *Apc*^*+/1638N*^ mice. All animal experiments were conducted in compliance with European guidelines for the care and use of laboratory animals and were approved by the Bavarian Government (Regierung von Oberbayern, Az.55.2-1-54-2532-161-2017).

Six-eight weeks old *Apc*^*+/1638N*^ and C57BL/6 mice were orally gavaged twice within 72 hours with 2 × 10^8^ *H. pylori* PMSS1 (DSMZ No.105294). The cecum, fecal, and the intestinal wash samples were collected from mice at 12 and 24 weeks after infection for processing and downstream analysis (Figure 1a). To obtain the intestinal wash samples, we gently flushed the contents of the small intestine using 5 ml syringes filled with cold phosphate-buffered saline (PBS).

## VLP isolation and metagenomic sequencing

Three to five samples from each group were pooled to get sufficient viral DNA for sequencing. Samples were vortexed vigorously at 4°C overnight, then centrifuged at 4,000 × g for 30 minutes to separate VLPs from bacteria and biological matters. The supernatant containing VLPs was filtered through 0.22 μm filters (Merck Millipore, SLGPR33RS) to remove the remaining unwanted materials. The filtrate was further concentrated to 5 mL by 10 kDa Amicon® Ultra Centrifugal Filters (Merck Millipore, UFC900324) and then purified using CsCl density gradient centrifugation at 24,000 rpm, for 4 h at 4°C. The following concentrations of CsCl were used to create the gradient: 1.2, 1.4, 1.5, and 1.65 g/cm^3^. Densities ranging from 1.39 to 1.51 g/cm^3^ were collected and concentrated to achieve 100 μL of highly concentrated VLPs. The samples were subjected to DNA extraction as described by Ma *et al*.^25^ The final concentration and quality of viral DNA were determined using the Qubit dsDNA HS kit (Invitrogen, Q32854) and sequenced using the Illumina Novaseq 6000 platform by Novogene.

## Metagenome assembly and analysis

Viromic sequencing data were analyzed using the ViroProfiler pipeline.^65^ Briefly, raw reads were filtered with fastp (v0.23.1)^66^ to remove adaptors and regions with low quality. Reads showing nucleotide identity to the mammalian host were removed by mapping clean reads to a masked host reference genome^67^ using bbmap.sh.^68^ The host reference genome was downloaded from the NCBI genome database and viral sequences were downloaded from viral RefSeq and neighbor nucleotide records in the NCBI nucleotide database, before being shredded using shred.sh script.^68^ The shredded virus sequences were then mapped to the host reference genome using bbmap.sh script. Mapped regions were masked using bbmask.sh script^68^ to create a virus-free host genome database. Clean reads were assembled into contigs using metaSPAdes (v3.15.4).^69^ Contigs (>3kb) generated from different samples were combined into a contig library (cclib). Duplicated contigs were removed using seqkit^70^ and dedupe.sh.^68^

CheckV (v0.8.1)^71^ was used to extract viral regions from contigs that contain proviruses. To further reduce the redundancy of the contig library, we clustered contigs following the “rapid genome clustering based on pairwise ANI” protocol in CheckV, which clustered contigs that share more than 95% identity and 80% coverage. The longest contigs from each cluster were selected to create the non-redundant contig library (nrclib). VirSorter2 (v2.2.3)^72^ and VIBRANT^73^ were used for the identification of viral sequences in nrclib. MMseqs2 taxonomy module^74^ and vConTACT2 (v0.11.1)^75^ were used for taxonomy annotation of viral operational taxonomic units (vOTUs). ORFs were predicted using Prodigal (v2.6.3)^76^ for each vOTUs.

Relative abundance data were obtained by mapping clean reads to nrclib using Bowtie2,^77^ and coverage and sequencing depth of each vOTU were calculated using CoverM (v0.6.1) (https://github.com/wwood/CoverM). Transcripts per million (TPM) was used to normalize the abundance table. BACPHLIP (v0.9.6),^27^ Deephage (v1.0)^28^ and in-house software, Replidec^29^ were used to predict the replication cycle of each vOTU. DRAM-v^38^ was used to identify and annotate the AMGs in vOTUs and manually curated using the UniProtKB database. Hosts of vOTUs were predicted using iPHoP.^78^

## 16S rRNA gene amplicon identification

The 16S rRNA data was obtained from Ralser’s study.^12^ Overlapping paired-end reads were processed with the DADA2 pipeline^79^ using the open-source software QIIME 2 v.2020.112.2 (https://qiime2.org).^80^ Unique amplicon sequence variants (ASVs) were assigned a taxonomy and aligned to the SILVA reference database (v138).^81^

## Statistical analysis

The diversity() function in the vegan package was used to calculate alpha (Shannon and Chao1 index) and beta diversities and the adonis() function to run PERMANOVA (permutational multivariate analysis of variance). The significantly abundant vOTUs (Sig. vOTUs) in samples from *Apc*^*+/1638N*^ infected by *H. pylori* PMSS1 were identified using the analysis of the composition of microbiomes with bias correction (ANCOM-BC) in R (v1.0.2).^82^ The cutoff of the log (fold change) and adjusted p-value (Benjamini – Hochberg) were set at 2 and 0.05. Co-occurrence networks inference on clr-normalized abundances was performed using the Spearman’s correlation in corrplot (v0.84) and NetCoMi package (v1.0.3)^83^ and significant edges were selected using cor.mtest(). Community structures were estimated using greedy optimization of modularity. The cutoff of the correlation and the p-value in the network of intra-bacteriome was set at 0.3 and 0.05, while the network of phage-bacteria was at 0.8 and 0.001, respectively. The ggplot2 package and GraphPad Prism 9.0 were used for plotting the graphs.

## Supplementary Material

Supplemental Material

## Data Availability

Sequence data have been deposited with links to BioProject accession number PRJNA808836 (16S) and PRJNA938071 (viral metagenomes) in the NCBI BioProject database.
