# Supplementary figures and images for: Gut virome profiling identifies an association between temperate phages and colorectal cancer promoted by Helicobacter pylori infection
Source: Gut Microbes. 2023 Sep 25;15(2):2257291. doi: 10.1080/19490976.2023.2257291 (PMC10578192; doi:10.1080/19490976.2023.2257291)

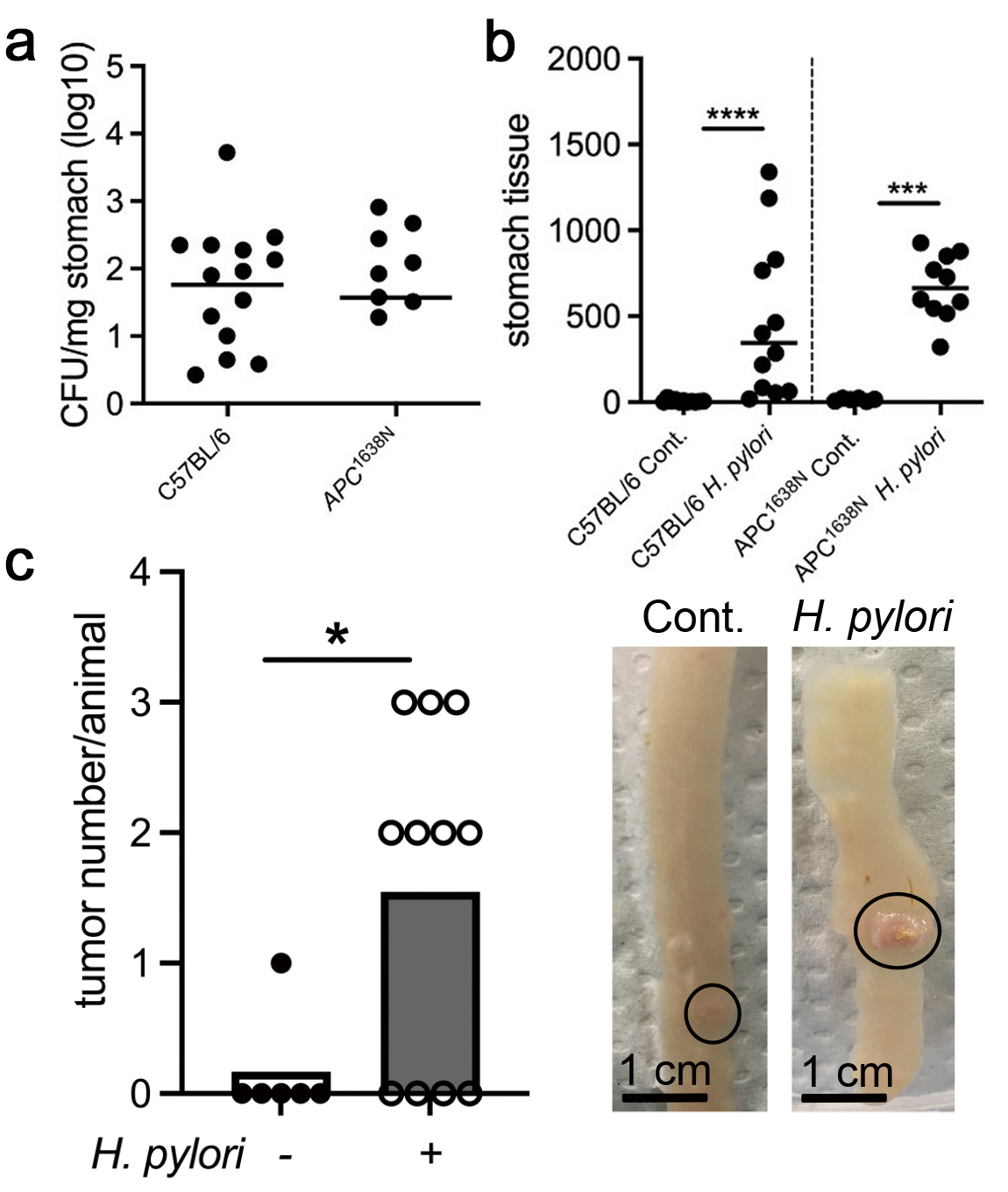

Supplement: Supplemental Material [file KGMI_A_2257291_SM6769.zip › KGMI_2257291_Supplementary material1/Supplementary Figure S1.jpg]

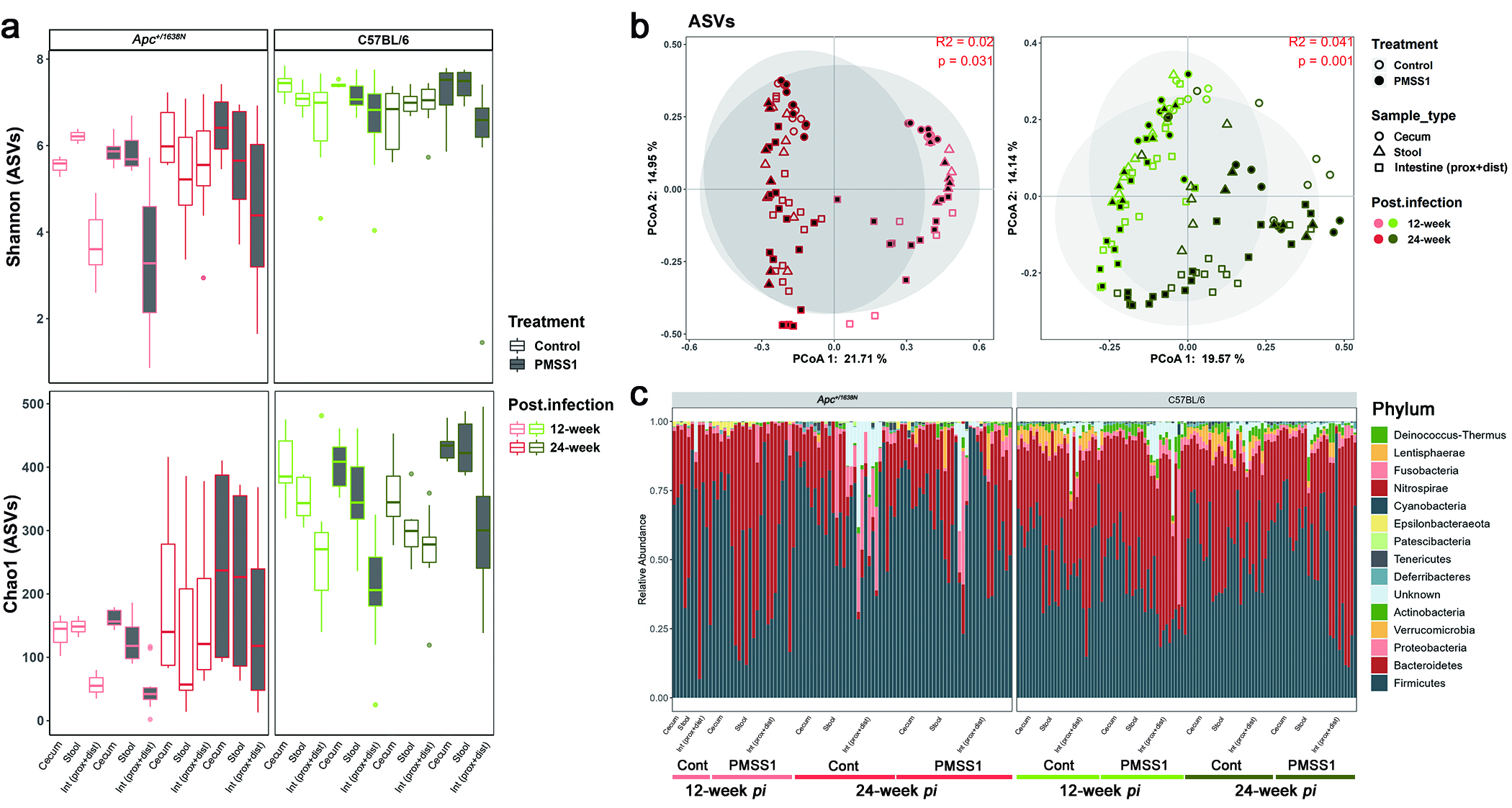

Supplement: Supplemental Material [file KGMI_A_2257291_SM6769.zip › KGMI_2257291_Supplementary material1/Supplementary Figure S2.jpg]

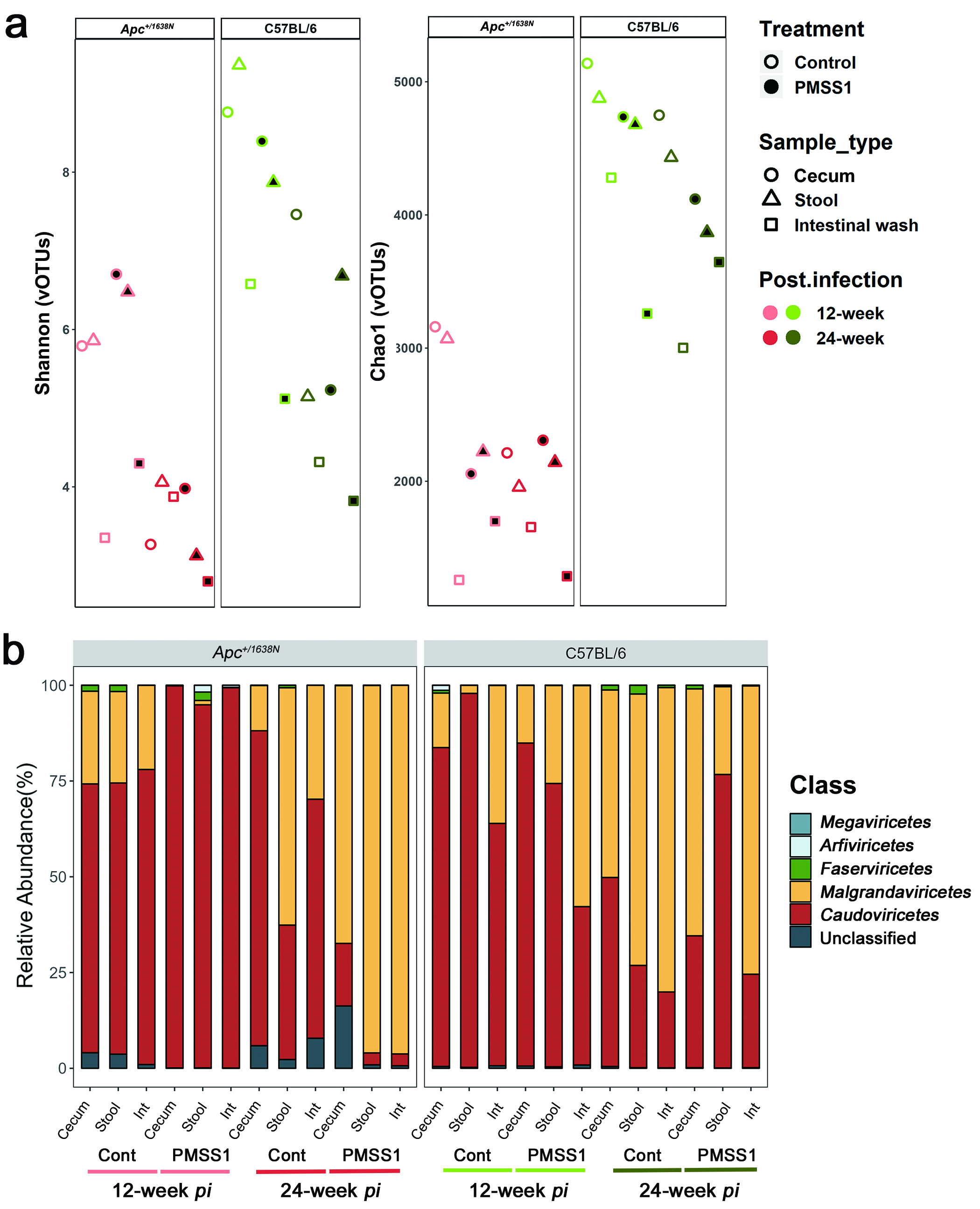

Supplement: Supplemental Material [file KGMI_A_2257291_SM6769.zip › KGMI_2257291_Supplementary material1/Supplementary Figure S3.jpg]

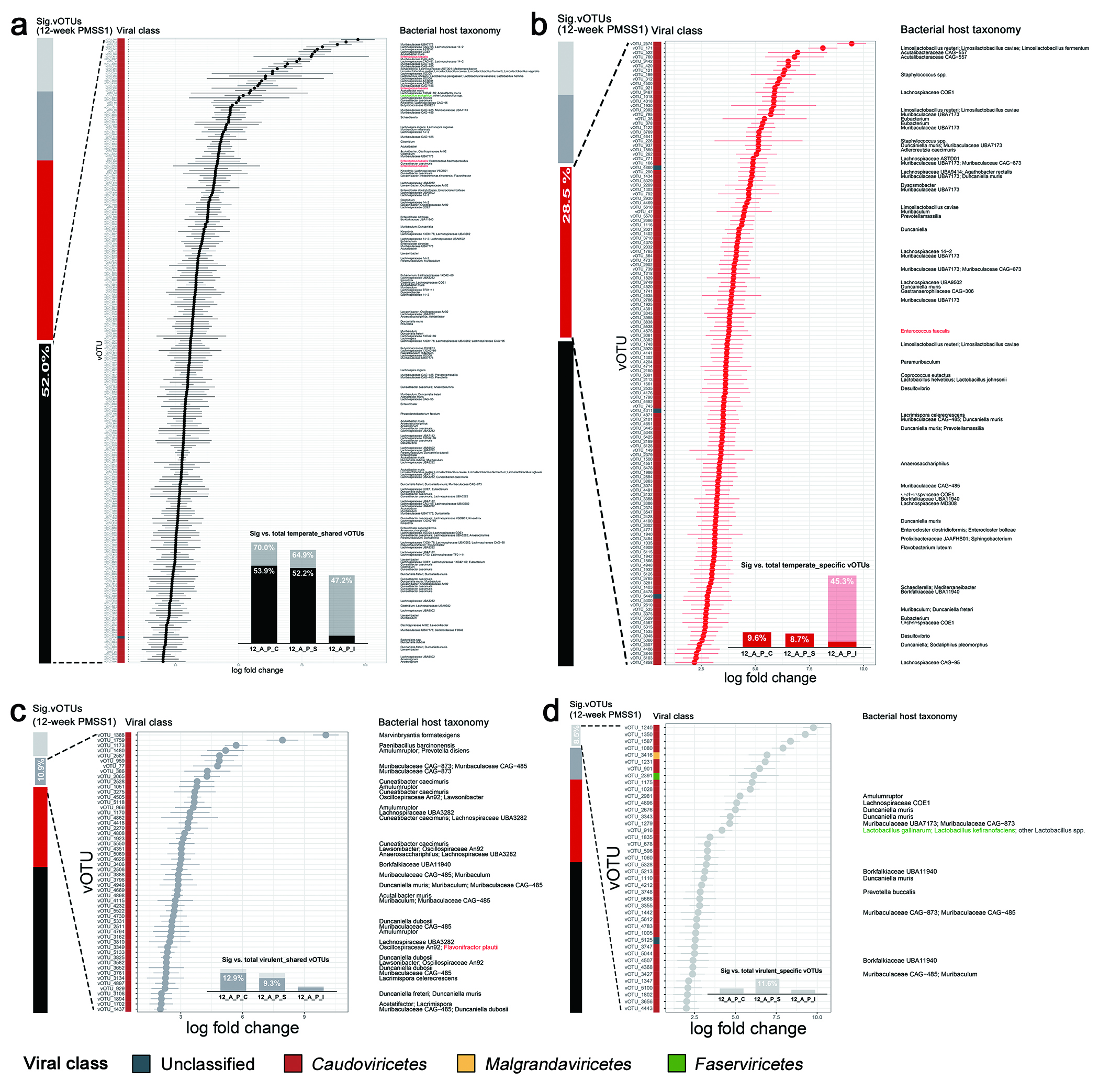

Supplement: Supplemental Material [file KGMI_A_2257291_SM6769.zip › KGMI_2257291_Supplementary material1/Supplementary Figure S4.jpg]

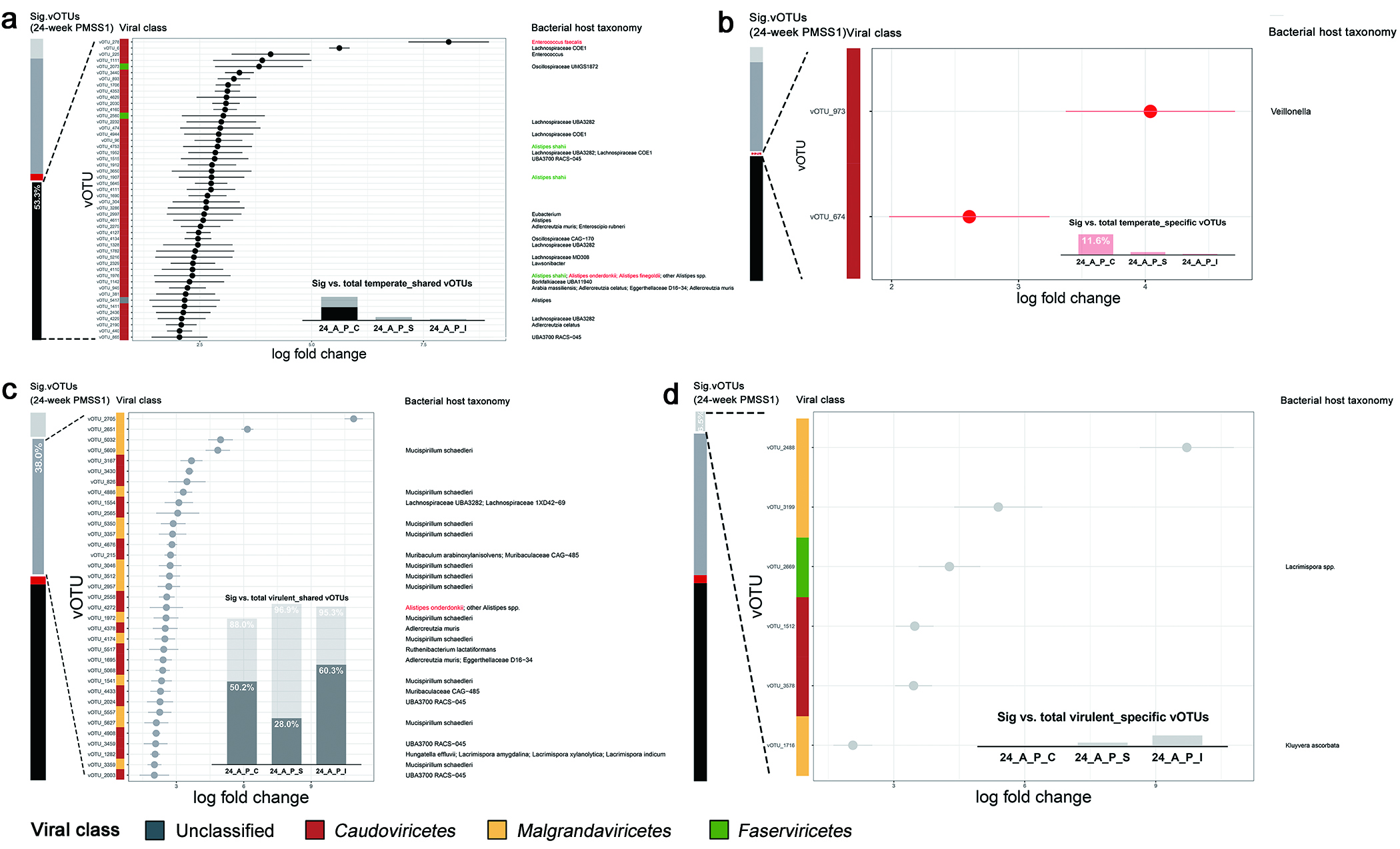

Supplement: Supplemental Material [file KGMI_A_2257291_SM6769.zip › KGMI_2257291_Supplementary material1/Supplementary Figure S5.jpg]

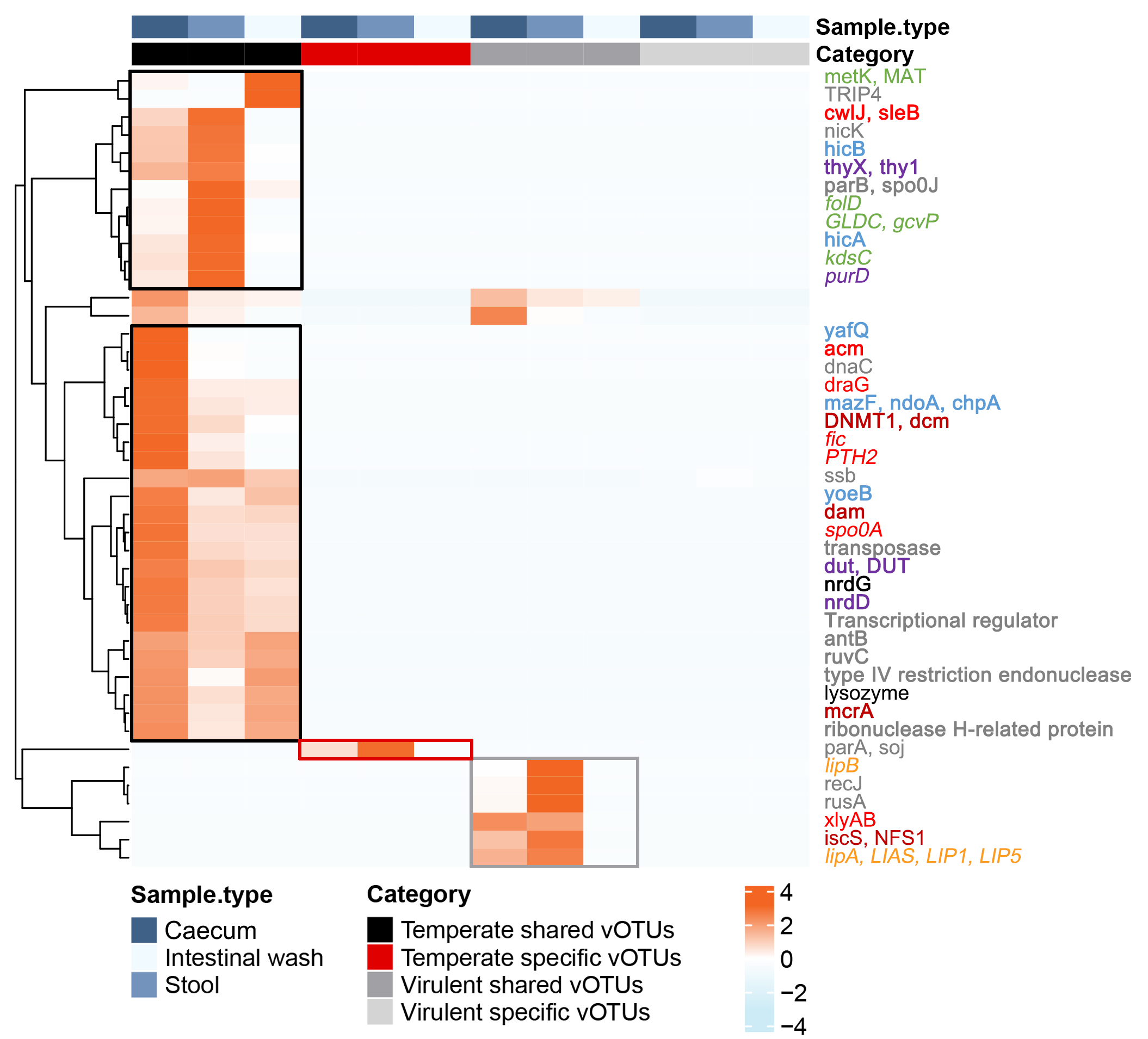

Supplement: Supplemental Material [file KGMI_A_2257291_SM6769.zip › KGMI_2257291_Supplementary material1/Supplementary Figure S6.jpg]

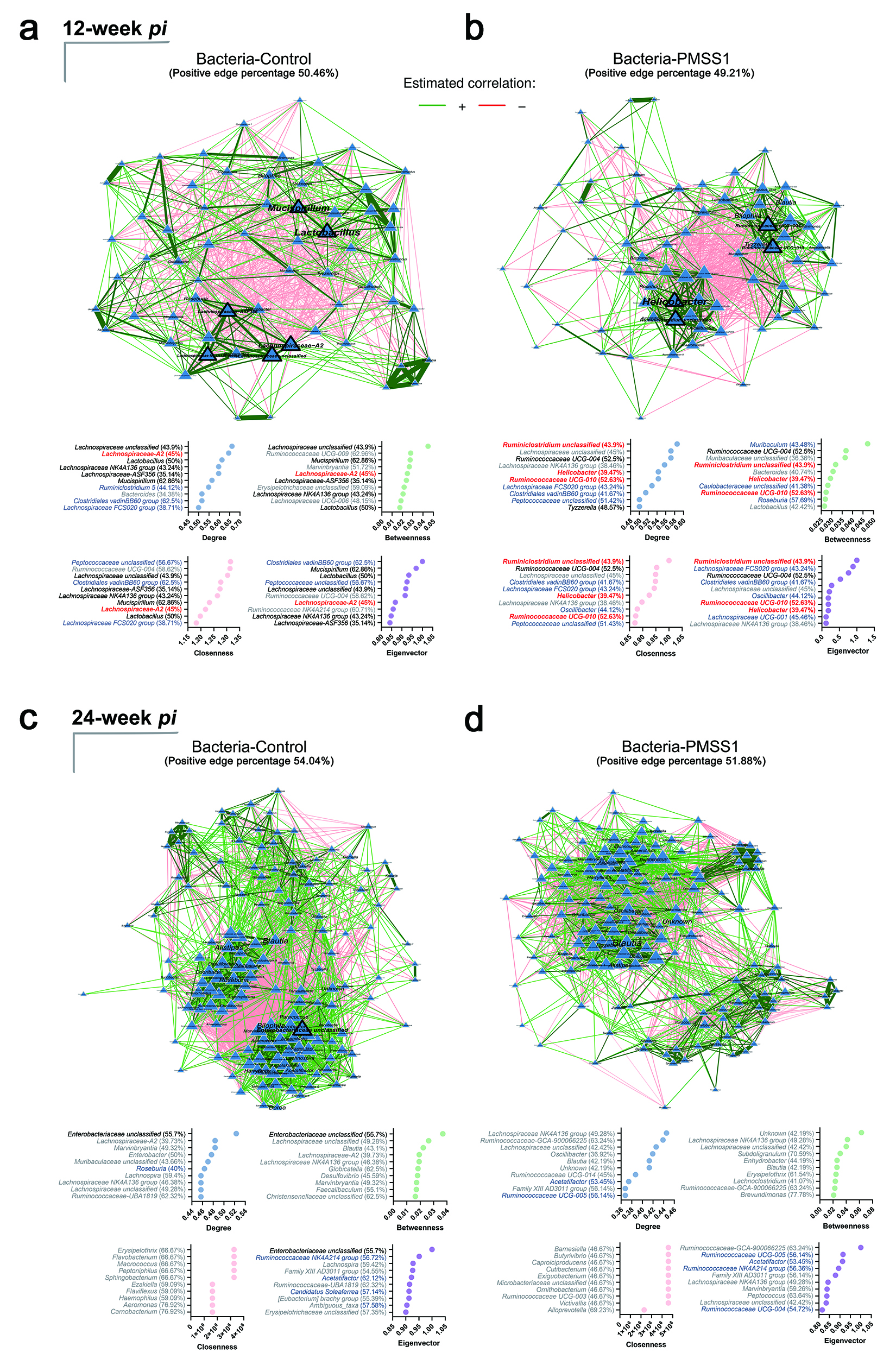

Supplement: Supplemental Material [file KGMI_A_2257291_SM6769.zip › KGMI_2257291_Supplementary material1/Supplementary Figure S7.jpg]

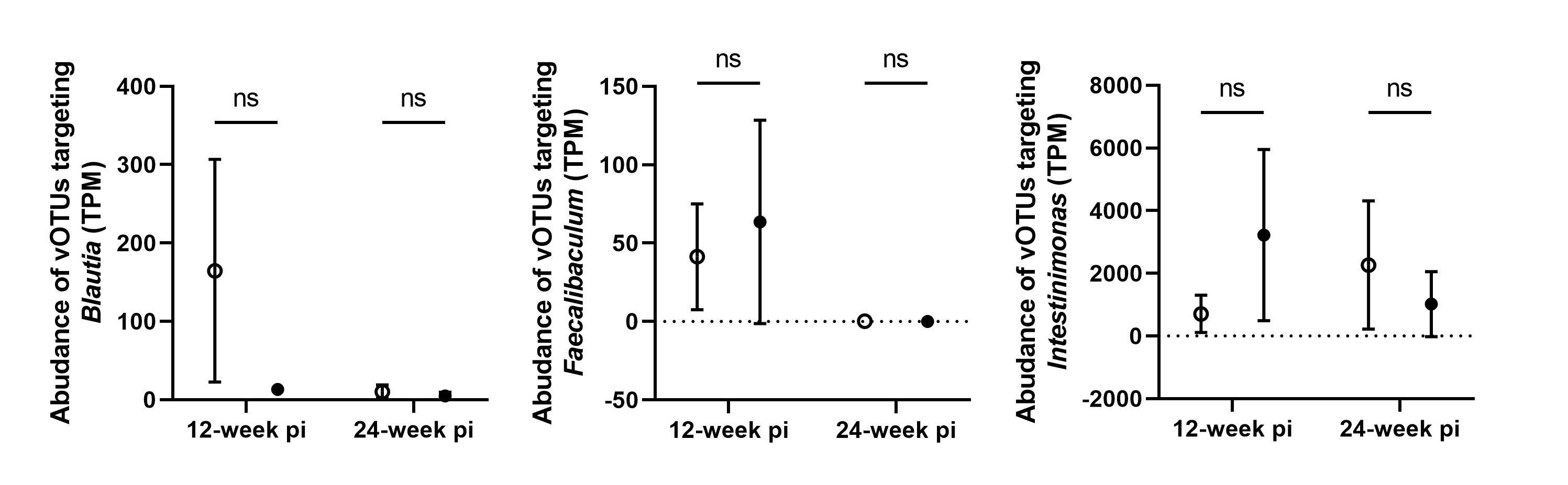

Supplement: Supplemental Material [file KGMI_A_2257291_SM6769.zip › KGMI_2257291_Supplementary material1/Supplementary Figure S8.jpg]
